# Therapeutic Efficacy of a Formulation Prepared with *Linum usitatissimum* L., *Plantago ovata* Forssk., and Honey on Uncomplicated Pelvic Inflammatory Disease Analyzed with Machine Learning Techniques

**DOI:** 10.3390/pharmaceutics15020643

**Published:** 2023-02-14

**Authors:** Sana Qayyum, Arshiya Sultana, Md Belal Bin Heyat, Khaleequr Rahman, Faijan Akhtar, Amin ul Haq, Batool Abdulelah Alkhamis, Mohammed Aedh Alqahtani, Reem M. Gahtani

**Affiliations:** 1 Department of Ilmul Qabalat wa Amraze Niswan, National Institute of Unani Medicine, Ministry of AYUSH, Government of India, Bengaluru 560091, Karnataka, India; 2 IoT Research Center, College of Computer Science and Software Engineering, Shenzhen University, Shenzhen 518060, China; 3 Centre for VLSI and Embedded System Technologies, International Institute of Information Technology, Hyderabad 500032, Telangana, India; 4 Department of Science and Engineering, Novel Global Community Educational Foundation, Hebersham, NSW 2770, Australia; 5 Department of Ilmul Saidla, National Institute of Unani Medicine, Ministry of AYUSH, Government of India, Bengaluru 560091, Karnataka, India; 6 School of Computer Science and Engineering, University of Electronic Science and Technology of China, Chengdu 610056, China; 7 Department of Medical Rehabilitation Sciences, College of Applied Medical Sciences, King Khalid University, Abha 62529, Saudi Arabia; 8 Department of Medical Laboratory Technology, Center for Poison Control and Medical Chemistry, King Khalid University, Abha 62529, Saudi Arabia; 9 Department of Clinical Laboratory Sciences, College of Applied Medical Sciences, King Khalid University, Abha 61421, Saudi Arabia

**Keywords:** inflammatory disease, honey, *L. usitatissimum* L., *P. ovata* Forssk., herbal medicine, AI for medicine, Unani system of medicine, bioactive molecule, oxidative stress, drug, machine learning

## Abstract

A single-blind double-dummy randomized study was conducted in diagnosed patients (n = 66) to compare the efficacy of Linseeds (*Linum usitatissimum* L.), Psyllium (*Plantago ovata* Forssk.), and honey in uncomplicated pelvic inflammatory disease (uPID) with standard drugs using experimental and computational analysis. The pessary group received placebo capsules orally twice daily plus a per vaginum cotton pessary of powder from linseeds and psyllium seeds, each weighing 3 gm, with honey (5 mL) at bedtime. The standard group received 100 mg of doxycycline twice daily and 400 mg of metronidazole TID orally plus a placebo cotton pessary per vaginum at bedtime for 14 days. The primary outcomes were clinical features of uPID (vaginal discharge, lower abdominal pain (LAP), low backache (LBA), and pelvic tenderness. The secondary outcomes included leucocytes (WBCs) in vaginal discharge on saline microscopy and the SF-12 health questionnaire. In addition, we also classified both (pessary and standard) groups using machine learning models such as Decision Tree (DT), Random Forest (RF), Logistic Regression (LR), and AdaBoost (AB). The pessary group showed a higher percentage reduction than the standard group in abnormal vaginal discharge (87.05% vs. 77.94%), Visual Analogue Scale (VAS)-LAP (80.57% vs. 77.09%), VAS-LBA (74.19% vs. 68.54%), McCormack pain scale (McPS) score for pelvic tenderness (75.39% vs. 67.81%), WBC count of vaginal discharge (87.09% vs. 83.41%) and improvement in SF-12 HRQoL score (94.25% vs. 86.81%). Additionally, our DT 5-fold model achieved the maximum accuracy (61.80%) in the classification. We propose that the pessary group is cost-effective, safer, and more effective as standard drugs for treating uPID and improving the HRQoL of women. Aucubin, Plantamajoside, Herbacetin, secoisolariciresinol diglucoside, Secoisolariciresinol Monoglucoside, and other various natural bioactive molecules of psyllium and linseeds have beneficial effects as they possess anti-inflammatory, antioxidant, antimicrobial, and immunomodulatory properties. The anticipated research work is be a better alternative treatment for genital infections.

## 1. Introduction

Pelvic inflammatory disease (PID) is an important gynaecological problem that is common in women of reproductive age living in developed or developing countries, with serious repercussions on their health and well-being [1,2]. It is an infectious and inflammatory disorder of the endometrium, fallopian tubes, ovaries, parametrium and peritoneum due to ascending infection of the endocervix, which presents varied patterns of polymicrobial infections and interrelated risks [3]. It is a common health issue in reproductive-age women in developed and developing countries [2]. Subclinical PID or uncomplicated pelvic inflammatory disease (uPID) can be defined as upper reproductive tract inflammation without any signs and symptoms of acute PID [4]. Uncomplicated PID must not be underestimated as it does not show clinical signs or symptoms, and it might destroy fallopian tubes as acute asymptomatic PID. Thus, asymptomatic sexually transmitted disease screening and early treatment are critical [4]. The CDC estimates that more than a million women experience an episode of PID every year [1]. Laparoscopic studies reveal that in 30–40% of cases, it is polymicrobial in aetiology. It leads to complications such as chronic pelvic pain, ectopic pregnancy, and infertility, and it increases health care costs [5]. The CDC has specified that for the clinical diagnosis of PID, at least one of the three minimum diagnostic criteria (viz., cervical motion tenderness or uterine tenderness/adnexal tenderness or lower abdominal pain) is a prerequisite. To increase the specificity of the minimum criteria, one or more of the following added criteria of PID are used to support the diagnosis: presence of a high number of WBCs in the vaginal fluid on saline microscopy, abnormal cervical or vaginal mucopurulent discharge, raised erythrocyte sedimentation rate, oral temperature >101 °F (>38.3 °C), raised C-reactive protein, and confirmation of *N. gonorrhoea* or *C. trachomatis* infection through laboratory diagnosis [1,2,6]. Uncomplicated PID is diagnosed on the basis of a clinical evaluation even though it has a lesser predictive value in comparison to laparoscopy [6]. The local signs and symptoms of uPID are copious, white or yellowish, and thin/thick discharge from the vagina with/without vulval itching, lower abdominal pain, and low backache during menses, menstrual disorders, dyspareunia, and infertility. Upon vaginal examination, eruptions are visualized very easily, there is tenderness on examination, and the uterine cervix is hypertrophied and can be palpated easily [7,8].

PID has developed as a silent killer that extensively disturbs women’s lives. The disease, if not treated, could result in acute morbidity and serious sequelae among women of reproductive age. The use of broad-spectrum antibiotics is the current centralized treatment according to guidelines [3]. Compliance is poor with antibiotic therapy for PID [4]. The most common and major side effects of antibiotics, analgesics, and anti-inflammatory drugs are headaches, gastrointestinal upsets, drowsiness, dizziness [9], renal impairment, and interactions with other medications [3]. Furthermore, an upsurge in multidrug resistant (MDR) pathogenic bacteria has been observed with a sharp reduction in conventional antibiotic therapy efficacy, which has increased the burden of disease in the population [10].

According to the World Health Organization (WHO), identified plants and their bioactive molecules would be the best alternative source to treat diseases. Unani classical texts and pharmacopoeia are enriched with medicines useful in uPID, such as the combination of *Plantago ovata* and *Linum usitatissimum* and honey [11,12], as they possess *Muhallil al-Waram* (anti-inflammatory), *Musakkin* (analgesic), and *Dafi’-i-Ta’affun* (antiseptic) properties [11]. *Plantago ovata* Forssk. belongs to the Family Plantaginaceae. The herb is found in northwestern India, cultivated to a small extent in West Bengal, Karnataka, and Coromandel Coast [13]. It is a soft, hairy, and small stemless annual herb. In India, ten species have been recorded, of which *P. ovata* is an important plant for its seeds [14,15]. The common name of *P. ovata* is *Isapghol*, which is derived from two Persian words—‘*Isap,*’ meaning a horse ear, and ‘*Ghol,*’ referring to the characteristic shape of its seed. The procedure and time of collection for the seeds include the spikes harvested in March–April, when they turn red. Usually, harvesting is completed in the early morning when a little dew is present, which prevents seed shedding. The material collected is threshed and winnowed and then sieved repeatedly until the seed is clear. For their preservation and storage, including the seeds, they are further cleaned and allowed to dry and then stored in airtight containers in cool and dry places [13]. The seeds are smooth, rosy-white, boat-shaped, ovoid-oblong, and convex on one side and concave on the other side. The outer layer of the ovule of the seed fuses with the inner epidermis on the concave side and forms a thin white membrane—the seed coat [15]. The microscopic longitudinal section is an oblong, elliptical and transverse section of seed that is oval in outline. The transverse section, cut through on one end of the seed, shows a central core of the radicle surrounded by the endosperm, whereas the other end shows two fleshy cotyledons. The seed coat structure is simple. The epidermis of the testa is composed of polyhedral cells, the walls of which are thickened by secondary deposits—the source of mucilage. In between the epidermis and the albumin, a thin brownish layer is found. Albumin is formed from thick-walled cells that are rich in matters such as fixed oil and proteins. The cells of the embryo are parenchymatous and packed with aleurone grains. The physicochemical parameters show that the organic chemical constituents present in psyllium seeds are protein, tannin, glycosides, fixed oils, and carbohydrates. The inorganic constituents are iron, zinc, potassium, and sodium [13]. It consists of various phytoconstituents such as pentosans, aldobionic acid, arabinose, galacturonic acid, rhamnose, fixed oil, proteins, xylose, galactose, uronic acid, glycoside-aucubin, tannin, and an active principle resembling acetylcholine. It also contains amino acids including alanine, glutamic acid, glycine, leucine, valine, lysine, and tyrosine, and fatty acids such as oleic, linoleic, and palmitic acids [16]. Its seeds are useful in hypercholesterolemia, gastrointestinal mucous-membrane inflammatory conditions and the genitourinary tract [14]. Pharmacologically, psyllium has been proven to have antibacterial, analgesic, antipyretic, and anti-inflammatory properties, and nephroprotective and hepatoprotective properties [17] in mice. Other studies on *P. ovata* have also proven it to have antimicrobial [18,19], anti-inflammatory, antipyretic [20,21] and antioxidant activities [22]. The aucubin in the seeds is responsible for its antibacterial properties. Plantamajoside, a bioactive molecule present in *P. ovata,* has been proven to have antibacterial activities [23].

*Linum usitatissimum* L. belongs to the Linaceae family. It is cultivated throughout the plains of India and up to altitudes of 2000 m above sea level [24]. Its seeds are commonly known as linseeds. The seeds contain two embryos: a thin endosperm and an embryo axis [25]. The testa is the seed coat or true hull. The seeds are about 4–6 mm in length and 2–2.5 mm in width. They are brown, elongated, ovate, flattened, rounded at one end, and obliquely pointed at the other end [26]. The procedure and time of collection is at the end of June and before the capsules or bolls ripen; the plants are heaved by hand, made up into stocks and left to dry in the field. The seeds are separated after the rippling process by threshing and winnowing the fruits. The seeds that are obtained are preserved and stored after drying and stored in a cool and dry place in air-tight containers. The organic chemical constituents are glucoside, oil, mucilage, protein, and tannin, and the inorganic constituents include potassium, magnesium, calcium, and sodium [24]. The taxonomy of the physicochemical parameters is summarized in Table 1 and Table 2, respectively. It also contains lignans, secoisolariciresinol diglucoside, alpha-linolenic acid, omega-3 fat [27], 30–40% fixed oil, 6% mucilage, and 25% protein. The two major fractions of the seed polysaccharides are aneutralarabinoxylan (75%) and acidic rhamnogalacturonan (25%). Linseed also contains amino acids such as threonine, lysine, and tyrosine [28]. In addition, it also contains tocopherols α, β, and γ forms as well as niacin [29]. It also contains 30.41% insoluble fibres and 10.22% soluble fibres [30]. The seeds also contain cyanogenic glycosides (prussic acid) [31]. Secoisolariciresinol diglucoside (SDG) is a phytoestrogen known to possess many favourable actions on human health and also contains antiviral, antibacterial, and antifungal properties [32]. Its seeds have a cancer-preventative effect and are effective for atherosclerosis, hypercholesterolemia, and chronic renal diseases because of the presence of an active principle-lignin secoisolariciresinol diglycoside [14,31]. Linseeds have been proven to have antimicrobial [30,33,34,35], anti-inflammatory, analgesic, antipyretic [36], and antioxidant activities [30]. Similarly, the antimicrobial activity of *L. usitatissimum* is mostly attributed to flavonoids, phenolic acid, and lignans [34].

Through repeated digestion and regurgitation, honey is formed by foraging bees that gather flower nectar and process it. The enzymatic activities of the honey bee and its stomach’s acidic pH, together with diastase, invertase, and amylase, make a supersaturated aqueous solution composed of 80% sugars—mainly fructose and glucose, with minor amounts of maltose, sucrose, and other complex sugars. The most abundant peptides are defensin-1 and royal jelly protein isoforms, while the major enzymes include diastase (amylase), glucose oxidase, catalase, α-glucosidase, and acid phosphatase [37,38]. The attributable numerous bioactive properties of honey are due to the miscellaneous composition of honey [38]. The aforementioned enzymes as well as phenolic acids and flavonoids are related to its antioxidant activity [38].

Honey bioactive molecules include methylglyoxal, phenolics, royal jelly proteins (MRJPs), and oligosaccharides. In royal jelly, there are royalizing peptides, antimicrobial jellein MRJPs, and hydroxy-decenoic acid derivatives, notably 10-hydroxy-2-decenoic acid (10-HDA) [37]. Honey has anti-inflammatory, antimicrobial, and antioxidant properties, and therefore has a possible therapeutic role in the treatment of various diseases. The physicochemical parameters of honey are summarized in Table 3.

The main two bioactive molecules [44], polyphenols and flavonoids, act as antioxidants and are present in honey [38,45,46]. The taxonomy and physicochemical parameters of psyllium, linseeds, and honey are summarized in Table 1, Table 2 and Table 3. However, to date, these plant materials with honey have not been studied clinically to treat uPID, even though few studies related to complementary and alternative medicines have been conducted [9,47,48]. Hence, we evaluated and compared the efficacy of the vaginal application of a formulation prepared with *L. usitatissimum*, *P. ovata*, and honey [12] on uPID with standard drugs, doxycycline, and metronidazole. In addition, we also used the same machine learning models [49,50,51,52,53,54] including Decision Tree (DT) [55,56,57], Random Forest (RF) [58,59], Logistic Regression (LR), and AdaBoost (AB) with three cross-validation models including 2-fold, 5-fold, and 10-fold to classify the standard and pessary groups. The main contributions of this study are given below:

- The pessary of psyllium and linseed with honey is beneficial for PID but has not been validated to date.
- The pessary group was beneficial, safe, and cost-effective in curing uPID as they possess anti-inflammatory, antimicrobial, analgesic, and antioxidant activities as proven in in vitro and in vivo studies.
- The design of an automatic classification model based on the experimental analysis of the pessary and standard groups on uPID occurring in reproductive-age women.
- The design of a detection system based on medicine using a machine-learning classifier.

This paper is organized based on the CONSORT guidelines for randomized controlled trials, as are the introduction, materials and methods, results, discussion and conclusion of the study.

## 2. Materials and Methods

### 2.1. Study Design and Population

**Figure 1:**
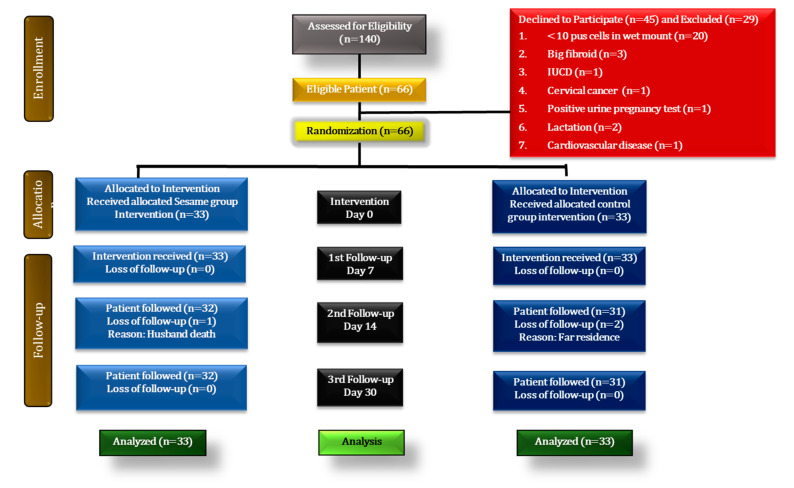
Consort statement for enrolment of participants.

This study was a prospective, double-dummy, single-centre, parallel, and single-blind randomized controlled trial (RCT) conducted at our hospital. A total of 140 participants were examined for uPID. Out of 140 women, 74 were excluded from the study after preliminary screening and investigations (Figure 1). Then, 66 participants were randomly allocated to the pessary (n = 33) and standard (n = 33) groups with a 10% dropout after fulfilling the inclusion criteria. The first participant was recruited on February 18, 2017, and the last follow-up was completed on 8 January 2018.

Married women aged 18–45 years who were diagnosed with any of the clinical features of uPID as per classical Unani texts [60] (abnormal vaginal discharge, adnexal and cervical motion tenderness, low backache, pelvic discomfort, dysuria, lower abdominal pain, dyspareunia) were included [7]. In addition, participants were also included based on presence of the one of the minimum criteria (lower abdominal pain, bilateral adnexal tenderness, and cervical motion tenderness) for diagnosis of PID as per Centre for Disease Control (CDC) guidelines. Participants with uPID were defined as having a vaginal mucopurulent yellow or green discharge, abnormal cervical discharge or observed on the cervix, and the presence of leucorrhea defined as WBC > 10/HPF in the saline microscopy of vaginal fluid as additional criteria [6,61].

Those excluded were pregnant or lactating women; women with pelvic or tubo-ovarian abscess and/or any problem expected to require surgical intervention within 24 h of the start of treatment; the use of systemic antibacterial therapy less than 7 days before enrolment; a history of cardiovascular abnormalities, epilepsy, or impaired liver and renal function; any history of pelvic, uterine or abdominal surgery ≤30 days before treatment; an inability or intolerance to an oral antibiotic regimen, and a delivery or abortion within the last three months.

### 2.2. Data Collection and Assessment Tools

#### 2.2.1. Data Collection

At the pre-study screening, we diagnosed women with uPID as shown by clinical features and one of the three minimum criteria from the CDC, abnormal vaginal discharge or cervicitis, and >10 WBC/HPF in the saline microscopy of vaginal fluid. At the baseline visit, relevant sociodemographic data, physical examination, and gynaecological examination were performed.

At baseline, the Visual Analogue Scale (VAS) for pain intensity to assess lower abdomen pain and lower back ache was used [61]. The VAS score is the most commonly used validated tool [48]. Participants were requested to mark on a colour-coding scale the VAS score that best described the actual status of their symptoms before the pelvic examination. The modified McCormack Pain Scale (McPS) was used to assess vaginal motion tenderness [47]. The amount of white discharge was estimated as absent (0), scanty (1) (normal moistness of the vagina and labia minora without staining or moistening the underclothes), moderate (2) (underclothes are soiled and require changing and washing frequently), or profuse (3) (requires the wearing of an extra pad, diaper or tampon) [62]. Clinical features such as dysuria, dyspareunia, and pruritus vulvae were considered present and relieved at baseline and follow-up. The SF-12 questionnaire was used to evaluate improvement in quality of life (QoL). The total Physical Composite Score (PCS) and Mental health Composite Score (MCS) of SF-12 HRQoL were calculated by utilizing the scores of twelve questions that ranged from 0 to 100; a score of 0 designated the lowest level of health, whereas 100 indicated the highest level of health.

Each patient was subjected to haematological biomarkers such as complete blood pressure, random blood sugar, erythrocyte sedimentation rate, and serological biomarkers, viz., HIV, VDRL, C-reactive protein, and complete urine analysis. The safety biochemical markers included blood urea, serum creatinine, phosphatase, aspartate transaminase, and alanine transaminase. Specific investigations such as pelvic ultrasonography, wet mount test (saline microscopy of vaginal discharge for WBC/HPF), and Pap smear were also performed to diagnose PID and its complications such as pelvic or tubo-ovarian abscess and cervical malignancy, respectively. A wet mount test was considered positive if WBC ≥ 10/HPF in vaginal discharge [48]. In known cases of controlled hypertension and hypothyroid participants, concomitant medication was continued as usual; however, the use of other concomitant medications that may have affected the outcome during the research period was not allowed.

#### 2.2.2. Assessment of Safety of Pessary and Standard Groups

The safety assessment included clinical features, examination and safety biomarkers of kidney and liver function tests to detect hepatotoxicity and nephrotoxicity at post-intervention (day 14). During follow-up, each participant was questioned regarding side effects such as local irritation, itching, and burning sensation after inserting the medicated tampon.

#### 2.2.3. Assessment of Haematological and Biochemical Markers

Blood from each participant was collected at the pre-screening visit. At room temperature, the collected blood was allowed to clot for 15–30 min, centrifuging the clotted blood at 3000–3500 rpm for 5–10 min separated the serum from it, and a valuation of biochemical markers was performed. Venous blood was collected in an EDTA tube for hemogram testing and was done by a COUNCELL V3 plus Automated haematology analyser. For the estimation of biochemical markers, a Chem 200 Technology Automated Biochemistry Analyzer was used. Glucose was estimated by the Glucose oxidase/peroxidase method [63]. ALT and AST levels were estimated by the UV-IFCC method [63]. The alkaline phosphatase level was estimated by 2-Amino-2-Methyl-1-propanol Buffer (IFCC) [64]. Serum urea level was valued by the Urease/Glutamate Dehydrogenase method [63]. Serum Creatinine level was estimated by the Picratte Method [65].

#### 2.2.4. Follow-Up Assessment and Withdrawal Criteria

Participants came for a follow up on day 7 and day 14 during the treatment period and one follow up on day 30 without treatment. They were also questioned in a phone call on day 3 and day 5 of treatment. At each visit, the change in clinical features, the McCormack pain scale, the VAS pain scale, and the HRQoL of the women were recorded. The participants who failed to follow the trial protocol and showed signs of any drug adverse reactions were withdrawn.

### 2.3. Intervention Used in Both Groups

#### 2.3.1. Selection and Identification

The Unani formulation for validation of uPID was carefully chosen from the Unani Pharmacopoeia [12]. Our institute’s pharmacy provided all trial-related medicines. The plant materials were directly procured from the local market by the institute’s pharmacy one week before the initiation of the trial. Then, these plant materials were sent for identification and authentication. We had procured certified honey from the Apiculture department, Gandhi Krishi Vigyana Kendra (GKVK), University of Agricultural Sciences, Government Institute, Bengaluru. The plant materials were identified as *Linum usitatissimum* L. whole seed and *Plantago ovata* Forsk whole seed at the Centre for Repository of Medicinal Resources, Trans-Disciplinary University, Bengaluru. For further reference, the specimens after identification were submitted to the institute with the voucher specimens (51/UQ/Res/2017).

#### 2.3.2. Method of Preparation, Dispensing, and Training of the Participants

**Figure 2:**
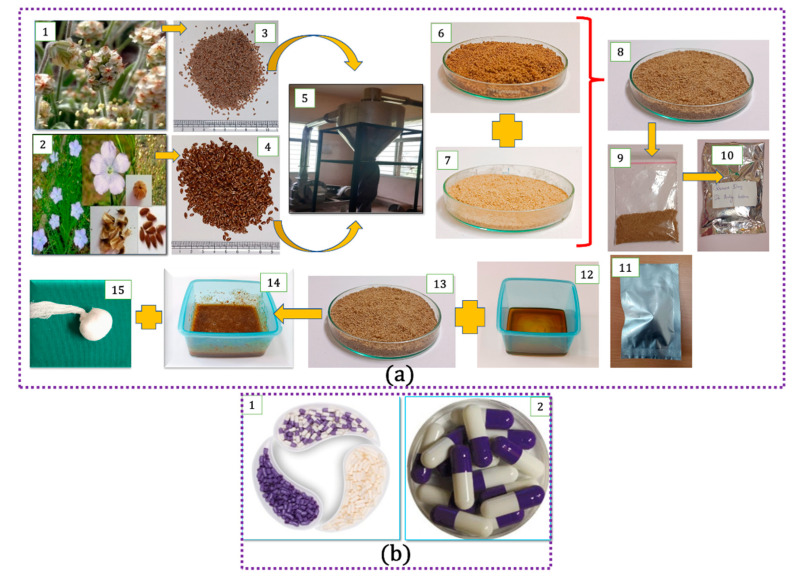
Depiction of pessary preparation: (**a**) (1) *Plantago ovata* plant; (2) *Linum usitatissimum* L. plant, seeds; (3) *Plantago ovata* plant, seeds; (4) *Linum usitatissimum L* plant, seeds; (5) powder-making machine; (6) powder of Linseeds; (7) powder of *P. ovata*; (8) mixed powder; (9) dispensing single dose in sachet; (10) sachets packed in the aluminium pouch; (11) dispensing of tampon; (12) honey; (13) mixed powder; (14) powder mixed with honey; (15) impregnation on tampon unfilled; and (**b**) (1) Unfilled and (2) filled placebo capsules.

Seeds were cleaned and powdered in a hammer mill separately and passed through Mesh No. 100. The powder was filled into small airtight pouches (Figure 2).

Each participant received 14 sachets in an aluminium pouch, each of which contained 6 gm of a mixed powder of both medicines and 70 mL of honey in a glass bottle. Each participant also received 14 sterilized cotton tampons individually packed in an aluminium pouch. A measuring container was also supplied to the participant. Each participant was trained on how to pour 5 mL of honey into a measuring container and add one sachet of powder in the container and mix it well. Then, she was advised to impregnate the tampon and insert it deeply into the vagina at bedtime and was advised to remove it in the morning. The capsules (500 mg) were filled with microcrystalline edible cellulose powder. Each participant received an individual pack containing 28 capsules dispensed in non-transparent aluminium pouches.

#### 2.3.3. Route of Administration and Dosage

Placebo: two capsules orally BID plus per vaginum tampon (cotton pessary/*hamul*) of fine powder of *L. usitatissimum* seeds (3 gm) and *Plantago ovata* seeds (3 gm) with honey (5 mL) at bedtime for 14 days was advised as per the traditional method in Unani classical text.

#### 2.3.4. Standard Controlled Group

For 14 days, each participant received doxycycline 100 mg twice daily and metronidazole 400 mg three times daily [66], as well as a placebo (palm sugar) vaginal tampon at bedtime.

### 2.4. Outcomes

For primary outcome measures, changes in clinical features (abnormal vaginal discharge and other symptoms), VAS pain scale for LAP and LBA and McCormack pain scale were assessed. The primary endpoint included a change in clinical response, defined as a 60% or greater reduction in vaginal discharge, VAS total score for pain intensity, and McPS score at day 14 when compared to baseline [61]. The percentage reduction (%R) score was calculated in Equation (1) below:

(1) $$\frac{\mathrm{Mean}\;\mathrm{score}\;\mathrm{on}\;\mathrm{day}\;14-\;\mathrm{Mean}\;\mathrm{score}\;\mathrm{on}\;\mathrm{day}\;0}{\mathrm{Mean}\;\mathrm{score}\;\mathrm{on}\;\mathrm{day}\;0}\times 100$$

For secondary outcomes, changes in WBC cells in saline microscopy of vaginal discharge from the wet mount test [61] and SF-12 health survey HRQoL questionnaire [67] were assessed. The secondary endpoint included clinical cure defined as WBC < 10/HPF in the saline microscopy of the vaginal discharge from a wet mount test and a 60% or more improvement in HRQoL.

### 2.5. Sample Size Estimation

The sample size estimation was carried out after a discussion with a biostatistician. The present study was of two groups, so the sample size was calculated around the mean difference of the pelvic tenderness score. The mean and SD were taken from the previous study mean (µ1 = 1.05 and µ2 = 0.50) and standard deviation (σ1 = 1.05 and σ2 = 0.82) [48]. The level of significance was *p* < 0.05. For a confidence interval of 95% and a power of the study of 90%, the following formula was used to calculate the sample size. As a result, it is calculated in Equations (2) and (3) below:

(2) $$n=2\;{\left(Z\alpha +Z\beta \right)}^{2}\times \;\sigma^{2}/\;{\left(\mu 1-\mu 2\right)}^{2}$$

(3) $$n=2\;{\left(1.96+1.28\right)}^{2}\times {\left(0.93\right)}^{2}/\;{\left(1.05-0.50\right)}^{2}=60.87$$

The sample size required in each group was 24. We included 33 participants in each group considering 10% dropout.

### 2.6. Randomization, Allocation, Blinding, and Treatment Adherence

Participants were randomly assigned into the pessary (n = 33) and standard (n = 33) groups after fulfilling the inclusion criteria. A computer-generated open list of a random number in a single block with a sequence of random assignments was used for simple randomization. However, the order of randomization was masked from the first researcher until the interventions were allocated to each participant. The double-dummy technique was used for masking and blinding. The medicine was dispensed in non-transparent aluminium pouches for masking. The participants were blinded. The participants were asked to bring back the empty pouches, and the number of unused sachets returned was counted and recorded on day 14 to check for compliance with the treatment. The data collection method for the sachet adherence measurement was similar to the pill count. If a participant failed to return dispensed sachets as a dropout, then it was assumed that sachets were not used and it was treated in the same way as returns. This variable was coded as 0% adherence if the participant dropped out of the treatment in the first post-randomization of the study and did not once return any sachets [68,69]. The formula used to calculate the compliance is in Equation (4) below:

(4) $$\frac{\left(\mathrm{Number}\;\mathrm{of}\;\mathrm{dosage}\;\mathrm{units}\;\mathrm{dispensed}\;-\;\mathrm{number}\;\mathrm{of}\;\mathrm{dosage}\;\mathrm{units}\;\mathrm{remained}\right)}{\left(\mathrm{prescribed}\;\mathrm{number}\;\mathrm{of}\;\mathrm{dosage}\;\mathrm{unit}\;\mathrm{per}\;\mathrm{day}\;\times \;\mathrm{number}\;\mathrm{of}\;\mathrm{days}\;\mathrm{between}\;2\;\mathrm{visits}\right)}$$

### 2.7. Statistical Methods

The statistical software SPSS 18.0 version 3.2.2 was used for data analysis. The results were presented in terms of mean ± SD for continuous variables and number (%) of categorical measurements. The alpha was 5% with a 95% confidence interval with a two-sided *p*-value. The Fisher’s or Chi-square test was applied for the comparison of the proportions wherever necessary. An intragroup comparison for normally distributed data, a paired Student’s *t*-test, and for skewed data, a Wilcoxon matched paired test was performed. The analysis included the student’s *t*-test (normally distributed data) and the Mann–Whitney *U* test (skewed data) for intergroup comparison. The intention-to-treat principle uses data from all randomized subjects with at least two post-randomization outcome measures for all efficacy variables. The last observation carried forward method was performed to impute missing data. The significance level was described as *p* > 0.05 (not significant) and *p* < 0.001 (extremely significant).

## 3. Results

### 3.1. Sociodemographic Characteristics

The study groups were homogenous, with no statistically significant differences between the pessary and standard groups (*p* > 0.05) in terms of age (31.36 ± 5.65 vs. 30.79 ± 5.40 years), age of menarche (12.63 ± 6.25 vs. 12.47 ± 5.78 years), menstrual cycle (29.72 ± 3.99 vs. 28.54 ± 3.77 days), duration of menstrual flow (4.63 ± 1.13 vs. 4.75 ± 0.96 days), duration of marriage (12.63 ± 6.25 vs. 12.46 ± 5.39 years) and body mass index (27.14 ± 4.73 vs. 26.16 ± 5.39 Kg/m^2^). In the pessary and standard groups, 18 (54.5%) and 17 (51.5%) participants were from the upper or lower class, respectively. In pessary and standard groups, 12 (36.4%) vs. 8 (24.42%) and 3 (9.1%) vs. 5 (15.2%) participants were from the lower and upper middle classes, respectively.

### 3.2. Primary Outcomes

**Figure 3:**
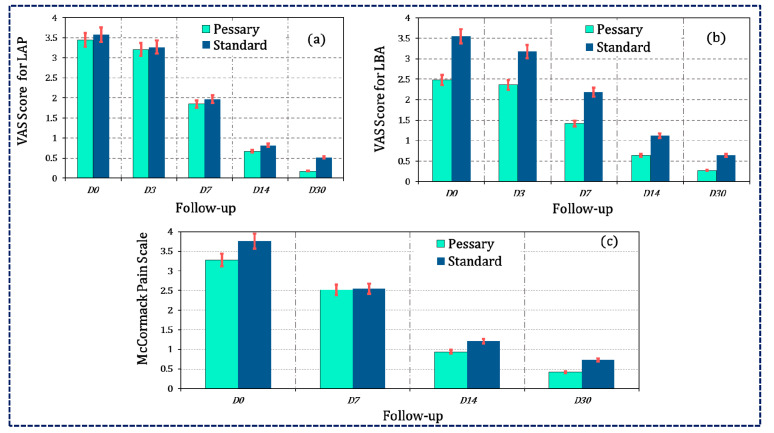
Graphical depiction of (**a**) VAS score for LAP, (**b**) VAS score for LBA, and (**c**) McCormack pain scale for pelvic tenderness.

Between the groups, visual analogue scale score, modified McCormack pain scale (Table 4 and Figure 3), and the comparison for clinical features (Table 5) were statistically insignificant, (*p* > 0.05) on day 0 and each follow up, whereas the intragroup comparison at day 14 compared to day 0 was statistically extremely significant (*p* < 0.001) in both the groups. The clinical response was more than 70% for all primary outcomes.

### 3.3. Secondary Outcomes

#### 3.3.1. WBC Count on Saline Microscopy of Vaginal Discharge

The WBC count in the wet mount in the pessary and standard groups on day 0 was 31 ± 11.14 and 36.36 ± 13.00/HPF on saline microscopy, respectively, and was statistically insignificant (*p* > 0.05), whereas on day 14, it was 4.69 ± 8.21 and 6.03 ± 8.97, respectively, and was also statistically insignificant (*p* > 0.05). The intragroup comparison was statistically significant with *p* < 0.0001 in both groups. The percent reduction in the pessary and standard groups on day 14 was 87.09% and 83.41%, respectively.

In the pessary group on day 0, 33.3% (n = 11/33), 30.3% (n = 10/33), 24.2% (n = 8/33), and 12.1% (n = 4/33) showed 11–20, 21–30, 31–40, and >40 WBC/HPF in the saline microscopy, respectively. On day 14, 45.5% (n = 15/33) had no WBC cells and 45.5% (n = 15/33) had 1–10 WBC/HPF, respectively. Three percent (n = 1/33) each showed 11–20, 21–30, and 31–40 WBC/HPF in the saline microscopy, with *p* < 0.0001 on day 14 from day 0. In the standard group on day 0, 15.2% (n = 5/33), 30.3% (n = 10/33), 30.3% (n = 10/33), and 24.2% (n = 8/33) had WBC/HPF values of 11–20, 21–30, 31–40, and > 40, respectively. On day 14, 0% (n = 0/33), 84.8% (n = 28/33), 6.1% (n = 2/33), 6.1% (n = 2/33), and 3% (n = 1/33) showed no WBC, 1–10, 11–20, 21–30, and 31–40 WBC/HPF, respectively, with *p* < 0.0001 from day 0.

#### 3.3.2. Heath-Related QoL Assessed by SF-12 Questionnaire

**Figure 4:**
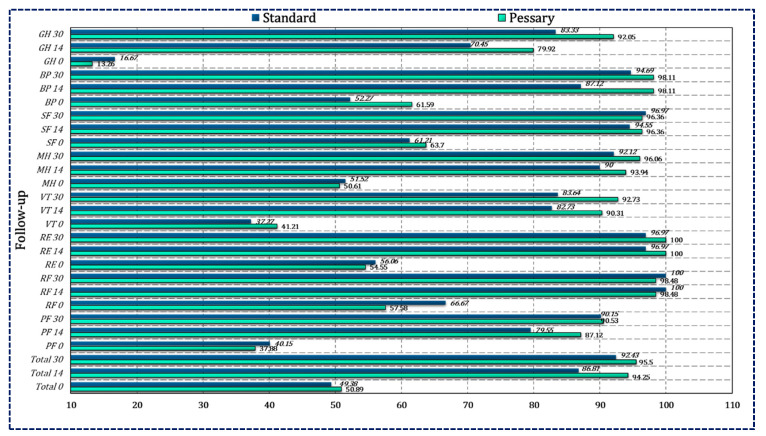
Comparative analysis between both standard and pessary groups based on SF-12 HRQoL at each follow-up.

The intragroup and intergroup comparison for the total score, eight domains of the SF-12, and the PCS and MCS scores were compared on days 0, 14 and 30. Table 6 summarizes the results of the SF-12 questionnaire. Between the groups, the comparison for vitality was statistically significant at day 30 (*p* < 0.037). The improvement of the SF-12 score was more than 70% in both groups (Figure 4).

### 3.4. Haematological and Biochemical Markers for Safety Parameters

None of the participants complained of any local irritation, itching, or burning sensation. The haematological and biochemical markers for the safety of the liver and kidneys and other investigations are summarized in Table 7. The statistical difference was found to be insignificant for all investigations before and after treatment. Further, none of the participants in either group reported any serious adverse events. In addition, Section 4 explains in detail the hepatoprotective and nephroprotective in vitro and in vivo research conducted for the safety of linseeds and psyllium.

### 3.5. Classification of the Pessary and Standard Groups using Machine Learning Techniques

For the classification, we used four machine learning techniques such as DT, RF, LR, and AB with three cross-validation models including 2-fold, 5-fold, and 10-fold to classify the pessary and standard groups (Table 8). The standard mathematical expression of the performance evaluation methods such as sensitivity (SENS), specificity (SPEC), accuracy (ACC), and precision (PREC) [70,71,72,73,74] are mentioned in Equations (5)–(8):

(5) $$\mathrm{SENS}=\frac{\mathrm{True}\;\mathrm{Positive}}{\mathrm{True}\;\mathrm{Positive}\;+\;\mathrm{False}\;\mathrm{Negative}}$$

(6) $$\mathrm{SPEC}=\;\frac{\mathrm{True}\;\mathrm{Negative}}{\mathrm{True}\;\mathrm{Negative}\;+\;\mathrm{False}\;\mathrm{Positive}}$$

(7) $$\mathrm{ACC}=\;\frac{\mathrm{True}\;\mathrm{Postive}\;+\;\mathrm{True}\;\mathrm{Negative}}{\mathrm{False}\;\mathrm{Postive}\;+\;\mathrm{False}\;\mathrm{Negative}\;+\mathrm{True}\;\mathrm{Negative}\;+\mathrm{True}\;\mathrm{Positive}}$$

(8) $$\mathrm{PREC}=\frac{\mathrm{True}\;\mathrm{Positive}}{\mathrm{True}\;\mathrm{Positive}\;+\mathrm{False}\;\mathrm{Positive}}$$

In the 2-fold model, DT achieved maximum performance in terms of sensitivity (57.40%), specificity (59.80%), accuracy (57.40%), and precision (55.70%). Additionally, LR achieved minimum performance in terms of sensitivity (57.50%), specificity (54.20%), accuracy (51.50%), and precision (50.00%). In the 5-fold, DT achieved maximum performance in terms of sensitivity (61.80%), specificity (63.90%), accuracy (61.80%), and precision (60.00%). Additionally, LR achieved minimum performance in terms of sensitivity (48.50%), specificity (51.50%), accuracy (48.50%), and precision (46.90%). In 10-fold, RF achieved maximum performance in terms of sensitivity (58.80%), specificity (61.20%), accuracy (58.80%), and precision (57.30%). Additionally, AB achieved minimum performance in terms of sensitivity (50.00%), specificity (54.20%), accuracy (50.00%), and precision (49.70%). The average performance was also found in terms of sensitivity (53.30%), specificity (56.63%), accuracy (53.30%), and precision (52.11%). However, our DT 5-fold model was better for classification in this study.

## 4. Discussion

### 4.1. Major Findings

The present study confirmed that a formulation prepared with linseeds, psyllium seeds, and honey was a safe and effective alternative herbal treatment to control uPID clinically. The clinical response for the primary and secondary outcome measures of the pessary group was above 70%. The observed effects of the formulation may be attributed to the synergistic effect of all individual ingredients. Overall, the compliance of the participant in the pessary and the standard groups were 98.46% and 96.92%, respectively.

### 4.2. Outcome Parameters

Concerning the primary outcome parameters, abnormal vaginal discharge is reported in thirty per cent of the population of India. Reproductive tract infections (RTIs) are a silent epidemic that distresses women’s lives [75,76] in developing nations. Intergroup comparison in the VAS score for LAP and LBA was not significant (*p* > 0.05) on day 14. However, the pessary group showed a higher percentage reduction than the standard group in abnormal vaginal discharge (87.05% vs. 77.94%), VAS-LAP (80.57% vs. 77.09%), LBA (74.19% vs. 68.54%), and McPS score (75.39% vs. 67.81%). In this study, the pessary group showed a reduction in vaginal discharge of 87.05%, a VAS score for LAP of 80.57% and 74.19% for LBA, and a McPS score similar to the findings reported in the previous studies [47,48,61]. Saeedi et al. [69] observed a noteworthy decrease in the symptoms. Similarly, another study carried out to evaluate the role of *Shivagutika* for 60 days in PID concluded that *Shivagutika* efficiently decreases the symptoms and clinically controlled the infection [9]. Another study also reported amelioration of abnormal vaginal discharge, backache, and other clinical features related to endo cervicitis [77].

In the secondary outcome parameters, the reduction of the WBC count in the saline microscopy was significant, with *p <* 0.001 in both groups from baseline, whereas between the groups, the comparison was statistically insignificant. However, the percent reduction of the WBC count was higher in the pessary than in the standard group (87.09% vs. 83.41%). In a previous study, the use of *Arq Mako* and *Sharbat Kasni* showed a 76.6% reduction in the WBC count [47], which was comparatively less effective than the present study. Health-related QoL improvement was seen in both groups. The Pap smear showed inflammatory cells in 21 (63.6%) participants before treatment; after treatment, the Pap smear was normal in 10 (48.5%) participants in the pessary group. Similar findings were reported in a previous study [78]. Studies have revealed that pelvic inflammatory disease is associated with bacterial vaginosis. The Pap smear finding showed that, in all participants, uPID associated with bacterial vaginosis was cured in the pessary group. Kahkashan et al. [79] also reported the beneficial effect of the Unani medicine *Pistacia integerrima* for bacterial vaginosis. On day 14, the SF-12 total score was 94.25 ± 5.80 in the pessary and 86.81 ± 13.14 in the standard group, with a significant difference (*p* < 0.004). The pessary group exhibited a meaningfully higher improvement in HRQoL than the standard group. Haggerty et al. [80], in their study, reported the women with chronic pelvic pain after PID had lower QoL. They concluded that chronic pelvic pain has a significant impact on physical and mental function, which worsens as pain intensity increases. They also discussed that women had lower scores for general health, bodily pain, vitality, physical functioning, social functioning, and mental health. This finding completely correlates with the present study. Previous human studies have revealed that inflammation plays a significant role in pelvic inflammatory disease progression [81]. Studies have reported that *L. usitatissimum* has analgesic and anti-inflammatory properties [82]. The improvement in HRQoL can be accredited to the anti-inflammatory, antioxidant, analgesic [14] and immunomodulatory [83] properties of *P. ovata*. *L. usitatissimum* has analgesic [26] and antioxidant properties [27]. *L. usitatissimum* is well known for the presence of omega-3 fatty acids and vitamins, and has the highest source of dietary lignan [84], which helps in improving quality of life. It also has antidepressant and antipyretic properties [36].

### 4.3. Overall Interpretation

The amelioration of abnormal vaginal discharge, LAP, LBA, McPS, and the absence of pus cells in the wet mount was probably attributed to the traditional ethnomedicinal properties such as the anti-inflammatory (*Muhallil al-Waram*), antiseptic (*Dafi’-i-Ta’affun*) and analgesic (*Musakkin*) properties of *P. ovata*, *L. usitatissimum* and honey [11]. Further, *P. ovata* has *Qabid* (astringent), *Mudirr-i-Bawl* (diuretic) and *Mulayyin* (laxative) properties and is useful in inflammatory conditions of the genitourinary tract [14], and gonorrhoea [15]. Honey has anti-inflammatory, antioxidant, antimicrobial, and immunomodulatory activities [37].

### 4.4. Antimicrobial Activity of Bioactive Molecules of Linseeds, Psyllium, and Honey

A previous study revealed that the methanolic extract of *P. ovata* takes more significant action against Gram-positive bacteria [18]. Another study showed that the silver nanoparticles of *P. ovata* seed shows good antibacterial activity against *Streptococcus aureus* [19]. Fons et al. [85] reported that the *Plantago* species’ secondary bioactive metabolites, i.e., iridoids, phenol, sterols, polysaccharides, alkaloids, and coumarins, can be supplemented as drugs to treat human diseases. Furthermore, a range of biological activities such as wound healing activity [83], antioxidant [20], analgesic, anti-inflammatory, immune-modulating, and anti-ulcerogenic activities of *P. ovata* have been proven. The plantamajoside is a caffeic acid derivative mainly credited for its antibacterial activity [23].

*L. usitatissimum* has anti-inflammatory, analgesic, diuretic, and laxative properties [11]. The mucilaginous infusion “Linseed tea” is useful for gonococcal infection [15]. Various pharmacological studies have proven its analgesic, antipyretic [36], Gram-negative and Gram-positive antibacterial properties [32,86]. One study screened its inhibitory effects against four types of Gram-positive and -negative bacteria, namely *S. aureus*, *B. cereus*, *K. pneumoniae*, and *P. aeruginosa,* for four different extracts of *L. usitatissimum* seeds. The petroleum ether extract established considerable inhibitory effects against all four bacteria. The ethanol extract also showed significant antibacterial activities [26,34,86]. Linseed protein extract showed antibacterial activity against the selected micro-organisms, especially Gram-negative bacteria [35]. Another study similarly stated the high antibacterial properties of linseed extract against *E. coli* and *S. aureus* [35].

In a previous study, Bodhankar et al. [10] assessed twelve medicinal plants for antimicrobial activity against selected pathogens as a substitute treatment for PID. They concluded that the active components exhibited antimicrobial activity against tested pathogens (*Streptococci* species, *S. aureus*, *Klebsiella*, *Salmonella* and *Candida albicans*), and the plants’ bioactive compounds included flavonoids, saponins, terpenoids, alkaloids, phenols and fixed oil. Similarly, the antimicrobial activity of *L. usitatissimum* is mostly attributed to flavonoids, phenolic acid, and lignans [34].

Honey can kill various bacterial strains as it contains a very high content of methylglyoxal (MGO), equal to about 1500 mg/kg. In addition, it acts against methicillin-resistant *S. aureus*. Nevertheless, honey also contains various other unidentified compounds that have potential action and antibacterial activity. A study has shown that MGO acts against *S. aureus* and *B. subtilis*. Other antibacterial factors isolated from honey are glycoproteins with high-mannose N-glycans [37]. Honey’s enzymatic glucose oxidation reaction and some of its physical aspects are the main sources of antimicrobial activity. In addition, the antimicrobial activity of honey’s other factors includes high osmotic pressure/low WA, low protein content, low pH/acidic environment, low redox potential due to the high level of reducing sugars, a viscosity that limits dissolved oxygen and other chemical agents/phytochemicals [45]. Glucose oxidase, hydrogen peroxide, low WA and water acidity are important properties of honey that do not help in the growth of yeast and bacteria [87]. Molan [88] reported that *Escherichia coli* and *Staphylococcus aureus* can be significantly prevented by manuka honey. It has been illustrated that the antibacterial activity of honey is effective on many bacterial pathogens and fungi [45].

### 4.5. Inflammatory Process in PID and Anti-Inflammatory and Antioxidant Activities of Linseeds, Psyllium, and Honey

Psyllium seeds and linseeds have analgesic, antipyretic, and anti-inflammatory properties [11,17]; hence, they were perhaps able to reduce pus cells in a vaginal wet mount. Pelvic discomfort depreciates the QoL and is a major and annoying issue for participants as well as physicians.

The inflammatory and idiopathic biological processes and the psychogenic, neuropathic, mixed and nociceptive processes are various causes of pelvic pain disorders. Tissue damage as the body’s reaction causes inflammatory pain, and the consequent inflammatory procedure activates “silent nociceptors” that usually don’t respond to thermal or mechanical stimuli. The essential causes of any type of pain are inflammation and their inflammatory response, which are mediated by inflammatory bio mediators (growth factors, neurotransmitters, neuropeptides, and cytokines). An injury to soft tissue or nerves may lead to the release of these inflammatory mediators and stimulates peripheral terminals of sensory nerve fibres, and released inflammatory neuropeptides are activated by the reverse firing of these sensory nerves. Inflammatory neuropeptides promote vascular permeability and vasodilation, and they recruit more immune cells (T helper cell) and triggers sensory nerve fibres in the surrounding area (neurogenic inflammation). Amplified input from peripheral pain receptors changes the central processing at the CNS level and modifies central processing mechanisms. In addition, local tissue inflammation also results in secondary hyperalgesia and central sensitization. Therefore, this can result in a syndrome that includes fever, joint pain, diffuse muscle pain, spasms, lethargy, and anorexia [89]. In response to the bacterial infection of *N. gonorrhoea*, an in vitro study has proven that lipid mediators important for inflammatory hyper nociception are produced from dendritic (PGE2) cells and neutrophils (leukotriene B4). In acute pelvic inflammatory diseases, elevated levels of both mediators have been noted in the peritoneal fluid [90]. Cyclooxygenase 1 and 2 are produced from arachidonic acid that produces prostaglandins (PGs), and PGs play an essential role in causing pain and inflammation. Currently, for pain relief and to reduce inflammation, common drugs such as steroids and nonsteroids are beneficial [91]. Therefore, targeting these responses may help to manage pain relief [90]. The source of any type of pain is inflammation and the inflammatory response. Hence, pelvic inflammatory disease can also cause pain.

Kopaei [82] confirmed the analgesic and anti-inflammatory effects of linseeds in mice. Linseeds show dose-dependent analgesic activities similar in part to morphine and might be used as an analgesic and anti-inflammatory agent [82]. The extract had flavonoid, phenolic, and flavonol compounds with antioxidant potential. Morsi et al. [92] estimated the total phenolic and flavonoid contents and also investigated the anti-inflammatory, antioxidant, and anticancer activities of various linseed extracts. The methanolic extract exhibited higher antioxidant activity. The GC-MS analysis displayed that the methanolic extract, ethyl acetate, and butanol fractions had 32, 40, and 36 compounds, respectively [92]. Mechchate et al. [93] investigated the anti-inflammatory and antidiabetic activity of a free polyphenol fraction of linseeds. Carrageenan is a chemical that helps the release of inflammatory mediators and was used in the experiment to induce acute inflammation. Carrageenan induced a biphasic response in the first hour after injection, inducing oedema formation and the release of histamine, serotonin and kinins. In the second phase, 2–3 h after the injection, it caused the release of PGs. PGs is a well-known cause of inflammation. The authors confirmed that the polyphenol fraction of linseeds had an anti-inflammatory effect in phase 1 and 2 of the carrageenan-induced inflammation [93]. The treatment with lignan extracts showed a significant reduction in malondialdehyde (MDA), whereas the levels of serum glutathione (GSH), glutathione peroxidase (GPx), catalase (CAT), superoxidase dismutase (SOD), zinc and manganese were significantly increased in paracetamol, with lignan-treated groups showing the antioxidant potential of lignans [94]. Khedher et al. determined the antioxidant and antiulcerogenic properties of psyllium seed ethanolic extract in rats and reported that during pre-treatment with psyllium, the oxidative stress status in the stomach tissues showed a significant increase in SOD, CAT, and GPx (antioxidant enzyme levels) with a significant decrease in lipid peroxidation. In conclusion, psyllium extract protects against gastric ulcers because of its antioxidant effect and the presence of bioactive molecules [21]. The phytochemical evaluation of the psyllium extract exhibited high numbers of flavonoids, polyphenols, and tannins possibly responsible for its anti-oxidative effect [95]. The above-mentioned compounds have crucial roles in quenching singlet and triplet oxygen or decomposing peroxides and absorbing and neutralizing free radicals [96]. Reddy et al. [83] confirmed the anti-inflammatory and antibacterial activities of psyllium.

The phenolic compound in honey has been an important contributor to its antioxidant capacity. The Italian multifloral honey extract contains major components, viz., the flavonoids, genistin, apigenin, luteolin, daidzein, kaempferol, quercetin, and chrysin. These compounds have inhibited the release of pro-inflammatory TNF-α and IL-1β. Immunomodulatory effects have also been reported for honey proteins [37]. Naturally, the level of enzymatic antioxidants such as SOD, CAT, GPx, and nonenzymatic antioxidants such as Vit. C and E decrease in the blood circulation and tissue as a result of an increase in inflammatory factors and damage. Hence, antioxidants have the potential to get rid of pain and inflammation [97]. Some plant compounds such as carotenoids, saponins, vitamin C, organic acids, alkaloids, tannins, anthocyanins, glycosides, vitamin E, and caffeic acid derivatives are effective in pain relief as they possess analgesic and anti-inflammatory effects [98]. Flavonoids have analgesic and anti-inflammatory activities [99] that are based on prostaglandin production inhibition in the central and peripheral system; flavonoids possibly prevent the metabolism of arachidonic acid.

Linseeds are traditionally used to ameliorate pain and inflammation. Linseeds include phenolic compounds, phenols, carotenoids, flavones, flavonoids, tannins, lignans, antocianins, vitamins E and C, and amino acids such as tyrosine, tryptophan, and proline. These plant bio compounds have diverse roles such as antioxidant activities [97]. Psyllium contains flavonoids and phenolics that have a reduced capacity and ROS scavenging activities. Psyllium seeds that had 78% polyunsaturated, 15% saturated, and 7% monounsaturated fatty acids were found [100] to contain flavonoids (natural polyphenol compounds) in their plants and have analgesic and anti-inflammatory effects [101]. These results are consistent with studies conducted on most medicinal compounds that have analgesic and anti-inflammatory properties. They prove their beneficial effects in reducing prostaglandins, their mediators, and enzymes that produce pain and inflammation as a significant peripheral factor in reducing the pain–inflammation process [102]. Further, lipoxygenase enzymes are related to various inflammatory-related diseases. Screening for the lipoxygenase (LOX)-inhibitory activities of linseed lignan extracts in various methods has been reported. Linseed extracts are also a potential source of novel therapeutics to treat many diseases related to the LOX enzyme [103].

TNF-α is a marker for inflammatory illness and it is a pro-inflammatory cytokine that has a critical role in modifying many physiological events as well as inflammation. *P. psyllium* extract successfully reduced CCl4-induced inflammation and hence TNF-α expression, indicating its healing effect on liver cells via the controlling of cytokine expression and the underlying inflammatory process [104]. Aucubin (a type of iridoid glycoside) and Lantamajoside exhibited anti-inflammatory properties through the inhibitory effect of TPA (12-o-tetradecanoylphorbol-13-acetate) [105] and arachidonic acid metabolism, respectively. Plantamajoside has an inhibitory effect on arachidonic acid metabolism, and therefore has anti-inflammatory [106] and also antioxidant properties [107]. Lantamajoside from psyllium has antioxidant and radical scavenging properties [83]. Tannins are recognized to ‘tan’ the mucosal outermost layer, interpreting it as less permeable and more resistant to injury or irritation [108]. The protective effects are associated with the antioxidant activities and antiradical power of psyllium extract [21]. The anti-inflammatory potential of honey is due to its phenolic content [109]. The pro-inflammatory activities of cyclooxygenase-2 and/or inducible nitric oxide synthase (iNOS) are suppressed by flavonoids and phenolic bioactive compounds [46]. Inducible nitric oxide synthase, tyrosine kinase, ornithine decarboxylase, and cyclooxygenase-2 are regulated by honey and its compounds. Numerous types of honey have been revealed to persuade tumour necrosis factor-alpha, IL-6 and interleukin-1 beta production. In addition, honey increases B and T lymphocytes, monocytes, neutrophils, eosinophils, antibodies, and natural killer cell generation in both primary and secondary immune responses in tissue culture [45].

### 4.6. Haematological and Biochemical Markers for Safety Parameters

The haematological and biochemical markers for the safety of blood, liver, and kidney showed no statistical difference before and after treatment and were within the normal range. This shows that both groups were safe on the liver, kidney, and blood, showing no toxicity of these medications in the body fluids and organs. It indicates that the research drugs were found to be safe. Further, clinically, the research drugs did not show any side effects during or after the completion of the trial. However, in the control group, the first three participants showed gastrointestinal and other symptoms such as pain in the epigastric region, vomiting, decreased appetite, headache, and giddiness after 2 days of ingestion of the standard drugs. Then onwards, all the participants were advised to take a tablet of pantoprazole 20 mg twice daily before eating food to avoid side effects. Khedher [21] investigated and tested animals that received different doses of Psyllium extract (100–1000 mg/kg). The data showed no acute toxicity or lethality up to 1000 mg/kg. Another study showed that animals who received psyllium extract at variable doses showed no signs of toxicity, morbidity or changes in animal behaviour. Analysis of serum renal and hepatic biomarkers showed that a dose of up to 1000 mg/kg of the extract was safe without any side effects [104].

### 4.7. Hepatoprotective and Nephroprotective Active of Psyllium and Linseeds

This section also explains in detail the hepatoprotective and nephroprotective in vitro and in vivo research of linseeds and psyllium. Previous studies have confirmed the hepatoprotective [17], immunomodulatory [83], and nephroprotective properties of *P. ovata* [15]. Further, the hepatoprotective and nephroprotective [110] properties of *L. usitatissimum* are also proven. Bagheri et al. [17] reported that an aqueous extract of psyllium seed showed hepatoprotective properties, and the tissue maintained normal architecture in the histological analysis of liver rats. They concluded that *Psyllium* seed extract was comparable to the standard hepatoprotective drug silymarin. They observed that indomethacin caused degenerative changes and cell necrosis in the liver’s cells and decreased hepatic microsomal cytochrome P-450 and prostaglandin [104]. Jumaily and Al Azawi [22] concluded that liver and renal damage from paracetamol-induced hepato-nephrotoxicity can be prevented, and pure lignan was better than partial lignan in rabbits. Another study [111] observed the hepatoprotective action of a linseeds chutney supplemented diet and was a probable source of antioxidants, and it might be used in protection against hepatotoxic damage induced by carbon tetrachloride (CCl4) in Wister rats.

### 4.8. Strength, Limitations and Future Recommendations

This was the first kind of study wherein a clinical intervention in which the ability of linseeds, psyllium and honey to reduce clinical features, VAS score, McCormack pain scale, and improve the HRQoL in uPID were investigated. Furthermore, it was a single-blind, prospective, parallel, randomized, controlled, and double-dummy study with intent-to-treat analysis and good patient compliance. Moreover, this study also included machine learning wherein an automatic classification model based on experimental analysis of the pessary and standard groups on uPID occurring in reproductive-age women was performed.

Due to the short duration of follow-up, specific diagnostic tests such as cervical swab culture, NAAT, and endometrial biopsy were not used to ascertain the microbiological and histological cure rates because of time restrictions and limited resources. Also, the data could not be generalized to all participants with acute PID, considering disease severity. Therefore, we suggest the aforementioned specific diagnostic tests for future studies. In addition, trial medicine might be researched in acute and complicated PID. This study was to validate the Unani pharmacopeial formula for its efficacy and safety. The authors also recommend carrying out the dosage form modification and standardization; the quantitative analysis of its bioactive molecules responsible for antimicrobial, anti-inflammatory and antioxidant activities; and a stability evaluation of the finished product. Additionally, the presence of active constituents in the bloodstream could be checked to assess their absorption and safety. Hence, elaborative pharmacokinetics and pharmacodynamics studies are recommended. Further, we would collect a large number of participants and design a hybrid model based on machine learning techniques to classify the drug and non-drug groups.

## 5. Conclusions

This data authenticates the claim of Unani scholars and is in consonance that the pessary group was as efficacious as the standard group for the treatment of uPID and thereby improved women’s HRQoL. Further, the treatment in the pessary group was a safe and economical alternative to antimicrobials to treat uPID. Healthcare providers can recommend the aforementioned Unani drugs to women to cure uPID. In addition, our DT machine learning model is more effective for the classification of both groups. Furthermore, double-blind RCTs and phase IV trials for a longer duration are recommended. In addition, well-designed studies with mechanisms need to be considered.

## Figures and Tables

**Table 1:** Taxonomy of psyllium and linseeds.

Taxonomy	Psyllium	Linseeds	Reference
Kingdom	Plantae	Plantae	[39,40]
Subkingdom	Viridiplantae	Viridiplantae	
Infrakingdom	Streptophyta	Streptophyta	
Superdivision	Embryophyta	Embryophyta	
Division	Tracheophyta	Tracheophyta	
Subdivision	Spermatophytina	Spermatophytina	
Class	Magnoliopsida	Magnoliopsida	
Superorder	Asteranae	Rosanae	
Order	Lamiales	Malpighiales	
Family	Plantaginaceae	Linaceae	
Genus	Plantago	Linum	
Species	*Plantago ovata* Forssk.	*Linum usitatissimum* L.	

**Table 2:** Physicochemical parameters of whole seeds of psyllium and linseeds.

Parameters	Psyllium	Linseeds	Reference
Ash value (%)			
Total ash	3.47	6.84	[13,24]
Acid insoluble ash	0.90	3.76	
Water soluble ash	1.10	0.41	
Successive extractive value (%)			
Petroleum ether	4.22	43.0	
Chloroform	1.40	1.1	
Ethanol	2.45	4.91	
Water	-	2.36	
Moisture content (%)	4.45	5.65	
Mucilage	22%	4.46%	[41,42]
Total phenolics mgGAE/g	286.8 ± 6.05	7.24 ± 0.14	[21,42]
Total flavonoids	101.83 ± 12.9 mgRE/g	1.74 ± 0.57 mg QE/g	[21,42]
Total tannins	39.33 ± 1.52 mgCE/g	1.35 ± 0.03	[21,42]

**Table 3:** Physicochemical parameters of honey.

Parameters	Values	Reference
Refractive index at 20 °C	1.485	[43]
Ash (%)	0.28	
Moisture (%)	20.83	
Total Soluble Solids (°Brix)	77.65	
P fund (mm)	103	
Colour	Light Amber	
Specific gravity	1.40	
pH	4.06	
Electrical conductivity (dS/m)	0.77	
Total reducing sugar (%)	69.36	
Sucrose (%)	2.94	
Glucose	33.77	
Fructose	35.59	
Hydroxy Methyl Furfural	27.30	

**Table 4:** The primary outcome measure based on VAS score for lower abdominal pain, lower backache (LBA), and Modified McCormack (McPS) for pelvic tenderness.

VAS for Lower Abdominal Pain	Pessary Group (n = 33)	Standard Group (n = 33)	*p*-Value
D0	3.45 ± 2.21	3.58 ± 2.65	0.841
D7	1.85 ± 1.46	1.97 ± 1.78	0.763
D14	0.67 ± 0.89 *	0.82 ± 1.33 **	0.589
D30	0.18 ± 0.46 ^*^	0.52 ± 1.09 **	0.112
%Pain Reduction (PR)	80.57%	77.09%	
VAS for lower back ache			
D0	2.48 ± 2.29	3.55 ± 2.69	0.090
D7	1.42 ± 1.44	2.18 ± 1.98	0.080
D14	0.64 ± 0.93 *	1.12 ± 1.47 **	0.115
D30	0.27 ± 0.63 *	0.64 ± 1.08 **	0.100
% Pain Reduction PR	74.19%	68.54%	
Modified McCormack Pain Scale (McPS) for pelvic tenderness			
D0	3.82 ± 1.59	3.76 ± 1.50	0.874
D7	2.52 ± 1.20	2.55 ± 1.00	0.912
D14	0.94 ± 1.00 *	1.21 ± 1.11 **	0.298
D30	0.42 ± 0.66 *	0.73 ± 1.01 **	0.154
% Pain Reduction (PR)	75.39%	67.81%	

* *p* < 0.001 and ** *p* < 0.001 on day 14 and day 30 from day 0 in the pessary group and standard group respectively; unpaired ‘t’ test and paired ‘t’ test; % PR: % pain reduction at day 14 compared to day 0.

**Table 5:** The primary outcome measures are based on abnormal vaginal discharge and associated symptoms of uPID.

Abnormal Vaginal Discharge (AVD)	Pessary Group (n = 33)				Standard Group (n = 33)				*p*-Value
Day 0	2.78 ± 0.41				2.72 ± 0.51				0.79
Day 14	0.36 ± 0.54				0.60 ± 0.86				0.39
*p* value	<0.0001				<0.0001				
% AVD reduction	87.05%				77.94%				
Associated symptoms	Present			Relieved	Present		Relieved		
Dyspareunia									
Day 0	21 (100)			0	18 (100)		0		
Day 14	3 (14.28)			18 (85.71)	4 (22.22)		14 (77.77)		0.41
*p* value	<0.0001				<0.0001				
Dysuria									
Day 0	10 (100)			0	10 (100)		0 (0)		
Day 14	0 (0)			10 (100)	2 (20)		8 (80)		0.23
Pruritus vulvae	0								
Day 0	23 (100)			0	19 (100)		0		
Day 14	0			23 (100)	3 (15.78)		16 (84.21)		0.08
AVD as per severity grading									
AVD Grading	0	1	2	3	0	1	2	3	--
Day 0	0	0	7 (21.2)	26 (78.8)	0	1 (3)	7 (21.2)	25 (75.8)	-
Day 14	22 (66.7)	10 (30.3)	1 (3)	0 *	19 (57.6)	10 (30.3)	2 (6.1)	2 (6.1) **	-
Day 30	28 (84.8)	5 (15.2)	0	0 *	25 (75.8)	5 (15.2)	2 (6.1)	1 (3) **	-

* *p* < 0.001 and ** *p* < 0.001 on day 14 and day 30 from day 0 in the pessary group and standard group respectively; unpaired ‘*t*’ test and paired ‘*t*’ test; % PR: % pain reduction at day 14 compared to day 0. Chi-square test and Fisher Exact test; AVD as per severity grading: 0—absent; 1—mild; 2—moderate and 3—profuse.

**Table 6:** The secondary outcome measure based on SF-12 HRQoL.

Domains SF-12 HRQoL	Period	Pessary Group (n = 33)	Standard Group (n = 33)	*p*-Value
Total score	Day 0	50.89 ± 23.38	49.38 ± 26.85	0.808
	Day 14	94.25 ± 5.80 ^a^	86.81 ± 13.14 ^b^	0.004
	Day 30	95.50 ± 6.86 ^a^	92.43 ± 11.88 ^b^	0.203
Physical function (PF)	Day 0	37.88 ± 35.42	40.15 ± 33.62	0.79
	Day 14	87.12 ± 19.64 ^a^	79.55 ± 19.22 ^b^	0.118
	Day 30	90.53 ± 18.50 ^a^	90.15 ± 18.69 ^b^	0.934
Role limitation due to Physical Problems (RF)	Day 0	57.58 ± 25.38	66.67 ± 27	0.164
	Day 14	98.48 ± 8.70 ^a^	100.00 ± 0.00 ^b^	0.321
	Day 30	98.48 ± 8.70 ^a^	100.00 ± 0.00 ^b^	0.321
Role limitation due to Emotional Problems (RE)	Day 0	54.55 ± 38.25	56.06 ± 39.05	0.874
	Day 14	100.00 ± 0.00 ^a^	96.97 ± 17.41 ^b^	0.321
	Day 30	100.00 ± 0.00 ^a^	96.97 ± 17.41 ^b^	0.321
Energy/Fatigue/Vitality (VT)	Day 0	41.21 ± 29.97	37.27 ± 33.66	0.617
	Day 14	90.30 ± 11.32 ^a^	82.73 ± 18.59 ^b^	0.05
	Day 30	92.73 ± 10.98 ^a^	83.64 ± 21.91 ^b^	0.037
Emotional well-being Mental Health (MH)	Day 0	50.61 ± 30.1	51.52 ± 32.32	0.906
	Day 14	93.94 ± 9.33 ^a^	90.00 ± 12.25 ^b^	0.147
	Day30	96.06 ± 8.99 ^a^	92.12 ± 12.19 ^b^	0.14
Social Functioning (SF)	Day 0	63.70 ± 33.69	61.21 ± 38.39	0.781
	Day 14	96.36 ± 12.45 ^a^	94.55 ± 9.05 ^b^	0.5
	Day 30	96.36 ± 12.45 ^a^	96.97 ± 7.28 ^b^	0.81
Bodily Pain (BP)	Day 0	61.59 ± 26.07	52.27 ± 28.89	0.174
	Day 14	98.11 ± 10.88 ^a^	87.12 ± 17.81 ^b^	0.004
	Day 30	98.11 ± 10.88 ^a^	94.69 ± 12.1 ^b^	0.39
General Health (GH)	Day 0	13.26 ± 16.52	16.67 ± 20.41	0.459
	Day 14	79.92 ± 18.73 ^a^	70.45 ± 19.22 ^b^	0.047
	Day30	92.05 ± 18.70 ^a^	83.33 ± 20.41 ^b^	0.075

Paired and Unpaired ‘*t*’ Test; ^a^ *p* < 0.001 and ^b^ *p* < 0.001 on day 14 and day 30 from day 0 in the pessary and standard groups, respectively.

**Table 7:** Haematological and biochemical markers in the pessary and standard group.

Variables	Period	Pessary Group (n = 33)	Standard Group (n = 33)	*p*-Value
Random Blood Sugar (mg %)	Day 0	99.76 ± 12.97	99.88 ± 14.74	0.972
Erythrocyte Sedimentation Rate (mm/1 h)	Day 0	35.85 ± 13.54	33.70 ± 14.59	0.537
	Day 14	30.91 ± 10.09 ^a^	35.36 ± 14.37 ^b^	0.15
Safety biomarkers				
Haemoglobin (Hb%) (gm%)	Day 0	11.35 ± 1.48	11.21 ± 1.15	0.665
	Day 14	11.17 ± 1.33 ^a^	11.26 ± 1.09 ^b^	0.779
Total Leucocyte Counts (cells/cumm)	Day 0	6515.15 ± 1439.21	6112.12 ± 1437.83	0.259
	Day 14	6309.09 ± 1673.20 ^a^	5975.75 ± 1267.4 ^b^	0.365
Aspartate aminotransferase (IU/L)	Day 0	22.28 ± 7.32	22.31 ± 9.52	0.988
	Day 14	21.33 ± 6.76 ^a^	22.24 ± 9.10 ^b^	0.647
Alanine transaminase (IU/L)	Day 0	24.62 ± 11.47	23.79 ± 11.3	0.769
	Day 14	23.76 ± 9.22 ^a^	24.97 ± 12.61 ^b^	0.657
Alkaline phosphatase (IU/L)	Day 0	199.60 ± 55.35	207.18 ± 42.04	0.533
	Day 14	204.52 ± 39.59 ^a^	202.27 ± 41.91 ^b^	0.824
Blood Urea (mg %)	Day 0	22.64 ± 5.63	23.12 ± 3.85	0.686
	Day 14	24.45 ± 3.59 ^a^	23.70 ± 4.21 ^b^	0.434
Serum Creatinine (mg %)	Day 0	0.76 ± 0.09	0.78 ± 0.10	0.52
	Day 14	0.76 ± 0.09 ^a^	0.75 ± 0.08 ^b^	0.579
Other investigations				
C-Reactive Protein				
Positive	Day 0	10 (100)	8 (100)	0.78
Negative	Day 14	5 (50)	5 (62.5)	1.00
Ultrasonography of abdomen				
Normal	-	16 (48.5)	21 (63.6)	0.32
PID		17 (48.5)	12 (36.4)	
P^H^ of vaginal discharge	Day 0	5.21 ± 1.05	4.70 ± 1.69	0.145
	Day 14	4.42 ± 0.71	3.48 ± 1.44	0.016
	*p* value	0.007	0.03	
Pap smear				
Normal		6 (18.2)	6 (18.2)	
Inflammatory	Day 0	21 (63.6)	24 (72.7)	0.548
Bacterial Vaginosis		6 (18.2)	3 (9.1)	
Normal		22 (66.7)	10 (30.3)	
Inflammatory	Day 14	11 (33.33)	22 (66.7)	0.003
Bacterial Vaginosis		(0)	1 (3)	
	*p* value	*p* < 0.001	0.365	

Fisher’s exact test; ^a^ *p* > 0.05 and ^b^ *p* > 0.05 on day 14 from day 0 in the pessary and standard group respectively.

**Table 8:** Classification of the pessary and standard groups based on different machine learning models.

Cross Validation-Fold	Classifier	SENS	SPEC	ACC	PREC
2	DT	57.40	59.80	**57.40**	55.70
	RF	55.90	58.40	55.90	54.20
	LR	57.50	54.20	51.50	50.00
	AB	48.50	56.80	48.50	48.50
**5**	**DT**	**61.80**	**63.90**	**61.80**	**60.00**
	RF	52.90	55.60	52.90	51.40
	LR	48.50	51.50	48.50	46.90
	AB	50.00	54.20	50.00	50.20
10	DT	51.50	54.20	51.50	50.30
	RF	58.80	61.20	58.80	57.30
	LR	52.90	55.60	52.90	51.20
	AB	50.00	54.20	50.00	49.70
mean		53.30	56.63	**53.30**	52.11
±SD		4.27	3.55	4.27	3.87

Bold number means highest classification performances.

## Data Availability

The data is available through the request via Prof. Dr. Arshiya Sultana (drarshiya@yahoo.com).
